# Acute progressive paravascular placoid neuroretinopathy with negative-type electroretinography in paraneoplastic retinopathy

**DOI:** 10.1007/s10633-017-9587-9

**Published:** 2017-04-05

**Authors:** Fred K. Chen, Avenell L. Chew, Dan Zhang, Shang-Chih Chen, Enid Chelva, Erandi Chandrasekera, Eleanor M. H. Koay, John Forrester, Samuel McLenachan

**Affiliations:** 1 Centre for Ophthalmology and Vision Science, The University of Western Australia, Perth, WA Australia; 2 Lions Eye Institute, Perth, WA Australia; 3 Department of Ophthalmology, Royal Perth Hospital, Perth, WA Australia; 4 Department of Medical Technology and Physics, Sir Charles Gairdner Hospital, Perth, WA Australia; 5 PathWest Laboratory Medicine WA, Fiona Stanley Hospital, Perth, WA Australia; 6 The Institute of Medical Sciences, The University of Aberdeen, Scotland, UK; 7 Ocular Tissue Engineering Laboratory, Lions Eye Institute, 2 Verdun Street, Nedlands, WA 6009 Australia

**Keywords:** Paraneoplastic retinopathy, Paravascular placoid neuroretinopathy, Cancer-associated retinopathy, Autoimmunity, Anti-retinal antibodies, Small cell carcinoma

## Abstract

**Purpose:**

Paraneoplastic retinopathy can be the first manifestation of systemic malignancy. A subset of paraneoplastic retinopathy is characterized by negative-type electroretinography (ERG) without fundus abnormality. Here we describe the multimodal imaging and clinico-pathological correlation of a unique case of acute progressive paravascular placoid neuroretinopathy with suspected retinal depolarizing bipolar cell dysfunction preceding the diagnosis of metastatic small cell carcinoma of the prostate.

**Methods:**

ERG was performed according to the International Society for Clinical Electrophysiology of Vision standards. Imaging modalities included near-infrared reflectance, blue-light autofluorescence, fluorescein and indocyanine green angiographies, spectral domain optical coherence tomography, ultra-widefield colour and green-light autofluorescence imaging, microperimetry and adaptive optics imaging. Patient serum was screened for anti-retinal antibodies using western blotting. Immunostaining and histological analyses were performed on sections from human retinal tissues and a patient prostate biopsy.

**Results:**

Serial multimodal retinal imaging, microperimetry and adaptive optics photography demonstrated a paravascular distribution of placoid lesions characterized by hyper-reflectivity within the outer nuclear layer resembling type 2 acute macular neuroretinopathy. There was no visible lesion within the inner nuclear layer despite electronegative-type ERG. Six months later, the patient presented with metastatic small cell carcinoma of the prostate. Tumour cells were immunopositive for glyceraldehyde-3-phosphate dehydrogenase, enolase and recoverin as well as neuroendocrine markers. The patient’s serum reacted to cytoplasmic and nuclear antigens in the prostate biopsy and in human retina. Anti-retinal antibodies against several antigens were detected by both commercial and in-house western blots.

**Conclusions:**

A spectrum of autoreactive anti-retinal antibodies is associated with a unique phenotype of acute progressive paravascular placoid neuroretinopathy resulting in degeneration of photoreceptor cells, inner retinal dysfunction and classic electronegative ERG in paraneoplastic retinopathy. Detailed clinical, functional and immunological phenotyping of paraneoplastic retinopathy illustrated the complex mechanism of paraneoplastic syndrome.

**Electronic supplementary material:**

The online version of this article (doi:10.1007/s10633-017-9587-9) contains supplementary material, which is available to authorized users.

## Introduction

Autoimmune retinopathy (AIR) can be paraneoplastic or non-paraneoplastic [1]. Expeditious and comprehensive systemic workup is critical for early detection of underlying malignancy in AIR patients presenting without a history of cancer. The diagnosis of AIR is often delayed due to its overlapping clinical phenotype with inherited retinal diseases and the lack of specificity of a positive anti-retinal antibody. Therefore, it is important to recognize the acute clinical features that are unique to paraneoplastic AIR to minimize delay in excluding malignancy.

Herein, we describe the multimodal imaging, electrophysiology, treatment response and immunological features of a case of acute paraneoplastic AIR characterized by an atypical macular neuroretinopathy, electronegative-type electroretinography (ERG) and a range of autoantibodies that react to both prostate cancer cells and several types of retinal neurons.

## Methods

### Patient recruitment

A 59-year-old Caucasian male presented in May 2015 and underwent full ophthalmic examination and automated visual field test. Informed consent was obtained for serial multimodal imaging and serum anti-retinal antibody analysis. The study protocol adhered to the tenets of the Declaration of Helsinki and was approved by the Human Research Ethics Office of the University of Western Australia (RA/4/1/7916).

### Clinical assessment

Imaging modalities included near-infrared reflectance (NIR), blue-light autofluorescence (AF), fluorescein and indocyanine green angiographies, spectral domain optical coherence tomography (SD-OCT, Spectralis HRA + OCT, Heidelberg Engineering, Heidelberg, Germany), ultra-widefield colour and green-light AF imaging (P200Tx, Optos plc, Dunfermline, UK), microperimetry (MAIA, CenterVue, Padova, Italy) and adaptive optics (AO) imaging (rtx1 camera, Imagine Eyes, Orsay, France). Electrophysiology was performed according to the International Society for Clinical Electrophysiology of Vision (ISCEV) standards (RETIport 3.2, Roland Consult, Brandenburg, Germany).

### Human ocular tissues

Human posterior eye cups were obtained from the Lions Eye Bank, Western Australia. The use of human tissue for research was approved by the University of Western Australia Human Research Ethics Office (Trial number: 2012-090).

### Human prostate histology

The tissue was fixed in formalin and embedded in paraffin, cut at 4 microns and submitted for routine hematoxylin and eosin staining. Immunohistochemistry was performed including appropriate positive and negative controls.

### Immunostaining

Paraffin-embedded human retinal or prostate tumour sections were heated at 50 °C for 1 h. The sections were then deparaffinized in xylene and rehydrated in graded series of ethanol. Heat epitope retrieval was performed in a rice cooker by boiling the deparaffinized sections for 20 min in antigen retrieval buffer (10 mM Sodium Citrate, 0.05% Tween20, pH 6). The sections in the buffer were then cooled on ice. After washing with TBST (50 mM Tris–HCl, 150 mM NaCl, 0.05% Tween20), sections were blocked for 1–2 h at room temperature with tris-buffered saline (TBS, 50 mM Tris–HCl, 150 mM NaCl) containing 10% normal goat serum and 1% BSA. The sections were then probed at 4 °C overnight with human serum (1:50), rabbit anti-recoverin (1:50), rabbit anti-synaptophysin (1:200), rabbit anti-Ki67 (1:200), mouse anti-GAPDH (1:100) or mouse anti-alpha-enolase (1:100) in TBS/1% BSA. After five TBS washes, sections were incubated with DAPI (1ug/ml) and goat antihuman AlexaFluor 488 (1:250), goat anti-rabbit AlexaFluor 488 or AlexaFluor 546 (1:250), anti-mouse AlexaFluor 546 (1:250) (Invitrogen), correspondingly. Negative controls were performed by omitting the primary antibody.

### Western blotting

Human retinal tissue was lysed in RIPA lysis buffer (Sigma-Aldrich, St. Louis, MO, USA) with 1% protein inhibitor cocktail (Sigma) followed by shaking at 4 °C for 1 h. The lysate was then centrifuged at 13,000 rpm for 30 min at 4 °C and the supernatant collected and stored at −20 °C. Protein concentration of the lysate was determined by Bio-Rad Protein Assay (Bio-Rad, Hercules, CA, USA). Human liver lysates were obtained from Santa Cruz Biotechnology (Dallas, TX, USA). Purified recombinant proteins were obtained from Abcam (Cambridge, England, UK, GAPDH and alpha-enolase) and Abnova (Taipei, Taiwan, PNMA2). Twenty micrograms of total protein from tissue lysates or 1 μg of a purified recombinant protein was mixed with Sample Buffer (Invitrogen, Thermo Fisher, Scientific, Waltham MA, USA) and heated at 70 °C for 10 min before loading onto a NuPAGE 4–12% Bis–Tris gel (Invitrogen). Protein samples were separated by gel electrophoresis in 1× MES buffer (Invitrogen) and then transferred onto a PVDF membrane (Immobilon-FL, Merck Millipore, Billerica, MA, USA) at 80 V for 90 min in transfer buffer (25 mM Tris, 190 mM glycine, 20% methanol, pH 8.3). After transfer, the membrane was incubated with Odyssey^®^ Blocking Buffer (LI-COR, Lincoln, NE, USA) at room temperature for 1 h. The membrane then was incubated with human serum (1:200) or rabbit anti-recoverin antibody (1:1000, ab85292, Abcam) in Odyssey^®^ Blocking Buffer plus 0.1% Tween20 at 4 °C overnight. After washing in TBST 3 times, the membrane was incubated with IRDye^®^ 800CW goat antihuman IgG (1:15,000; LI-COR) in Odyssey^®^ Blocking Buffer plus 0.1% Tween20 and 0.01% SDS for 1 h at room temperature. After 3 TBST washes, the membrane was washed once with TBS without Tween20. The membrane was then imaged at 800 nm wavelength by the Odyssey Infrared Imager (Model 9120, LI-COR).

## Results

The patient presented with a 3-month history of darkening of vision and delayed adaptation to reduced ambient light. He had no previous malignancy. Best-corrected visual acuity on the Early Treatment of Diabetic Retinopathy Study (ETDRS) chart was 87 and 85 letters in right and left eyes, respectively. Fundus examination revealed a trace amount of cells in the vitreous and cuffing of retinal veins adjacent to the discs in both eyes. Apart from mild pigment mottling of the retina, there was no discrete retinal lesion.

**Fig. 1:**
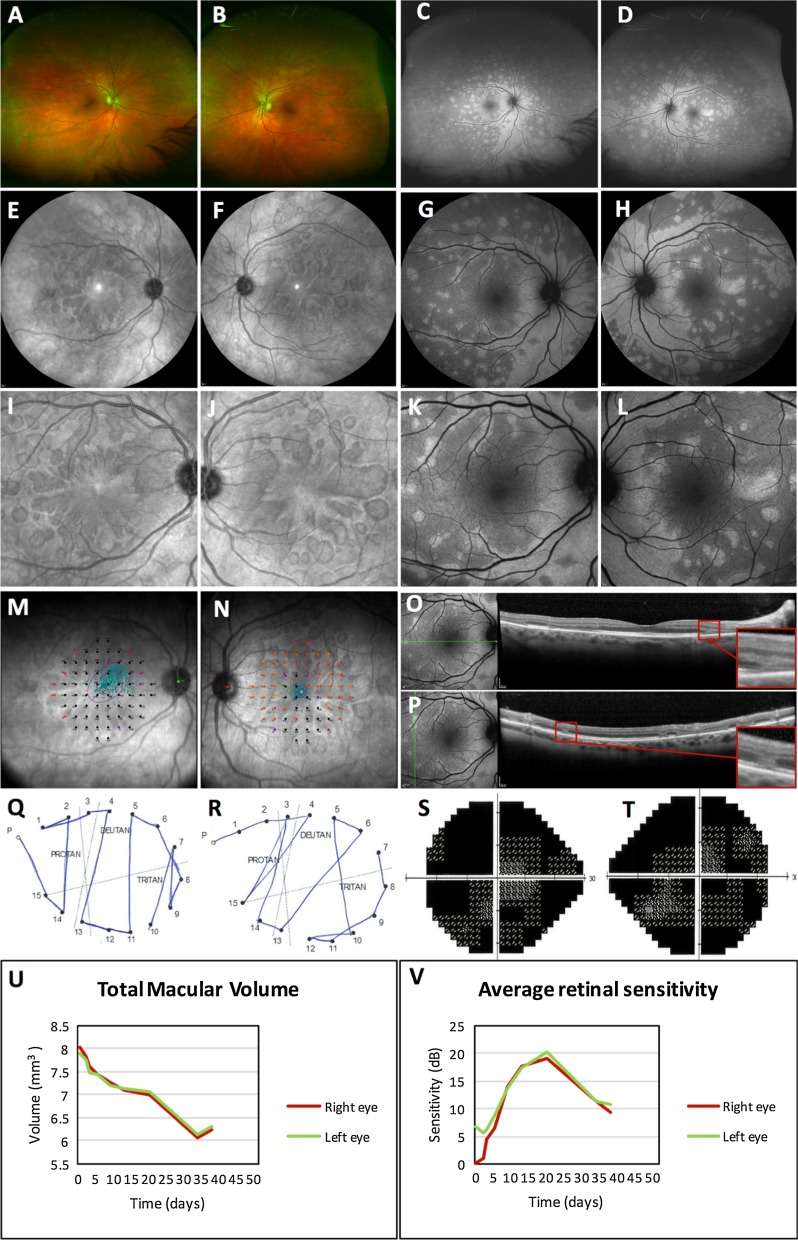
Retinal imaging and microperimetry. **a**, **b** Ultra-widefield *colour* photographs of the right and left eyes showed no discrete retinal lesions. **c**, **d** However, there was increased signal on green autofluorescence in the peripapillary region extending out along the major vessels. **e**–**j** Near-infrared reflectance imaging showed discrete hypo-reflective wedge-shaped lesions pointing towards the fovea in a petalloid pattern in both eyes. **g**–**l** Blue autofluorescence images revealed hyper-autofluorescence along the major vessels, sparing the central macula in both eyes. **m**, **n** Microperimetry revealed dense scotoma (represented by *black dots*) affecting the central 20° of the macula in both eyes. **o**, **p** There was photoreceptor layer loss and retinal thinning on OCT B-scans in the regions of increased autofluorescence due to unmasking of autofluorescence from the retinal pigment epithelium. *Insert* shows hyper-reflective outer nuclear layer at the margin of these lesions. **q**, **r** D-15 *colour vision* testing revealed colour deficiency along the deutan axis. **s**, **t** There was profound field defects in both eyes with 24-2 Humphrey visual field testing. **u**, **v** There was progressive reduction in total macular volume measured on OCT volume scans throughout the follow-up period despite steroid treatment, but retinal sensitivities on microperimetry did show considerable recovery after steroid treatment

Near-infrared reflectance (NIR) showed hypo-reflective lesions extending along vascular arcades and discrete wedge-shaped lesions pointing towards the fovea in a petalloid pattern. These lesions corresponded to severe thinning of the outer nuclear layer (ONL) and loss of the interdigitation zone (IDZ) surrounded by an adjacent region of hyper-reflectivity within the ONL (Fig. 1). Microperimetry showed dense scotoma even within the region of preserved IDZ and cone structures as shown by *en face* spectral domain optical coherence tomography (SD-OCT) and adaptive optics (AO) imaging (Online Resource 1). These placoid lesions had increased autofluorescence (AF) signal. Fluorescein angiography showed no specific features in the early phase. However, the paravascular and discrete placoid lesions became hyper-fluorescent in the late phase with segmental staining of the retinal veins. Indocyanine green angiography showed normal choroidal perfusion (Online Resource 2).

**Fig. 2:**
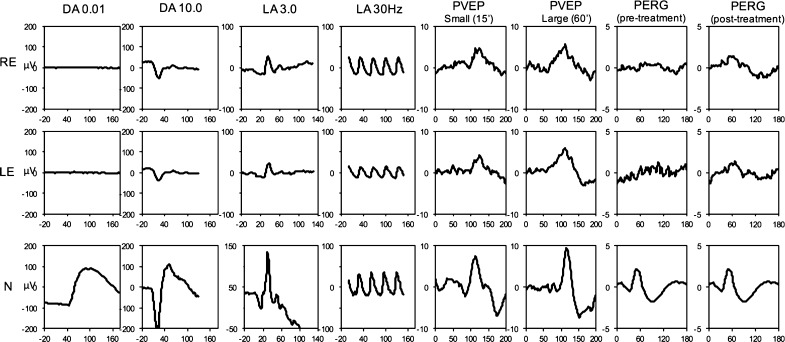
Electroretinography. Electrophysiology showed profound reduction in the rod-specific response (DA 0.01), reduced and delayed strong-flash a-wave (DA 10.0) and standard-flash photopic ERG (LA 3.0). 30 Hz flicker ERG (LA 30 Hz) was mildly delayed and reduced. There was reduced amplitude and increased latency in small check visual evoked potentials (PVEP small), but the large check visual evoked potentials (PVEP large) were within normal limits. Pattern electroretinogram (PERG) was essentially flat on presentation, with some recovery occurring post-steroid treatment

Electrophysiology showed a profoundly reduced rod-specific (dark adapted, 0.01 cd s/m^2^) response; an electronegative (b/a ratio of 0.8) combined rod-cone ERG, absent oscillatory potentials (dark adapted, 3.0 cd s/m^2^); reduced and delayed strong-flash a-wave (dark adapted, 10.0 cd s/m^2^); a mildly delayed and reduced 30-Hz flicker ERG; and a markedly delayed and reduced standard-flash photopic ERG (light adapted, 3.0 cd/m^2^) response characterized by a broadened trough and a sharply rising b-wave (Fig. 2). Pattern ERG was essentially flat. The large check visual evoked potential (VEP) was within normal limits, whereas small check VEP amplitude and latency were subnormal.

Based on symptoms and clinical and ERG features, AIR was suspected and serum was collected for western blot and immunohistochemistry (IHC) by a commercial laboratory (Ocular Immunology Laboratory, Casey Eye Institute OHSU, Portland, Oregon, US). Systemic workup including thoracic computed tomography (CT) and abdominal ultrasound showed no evidence of lymphadenopathy or tumour. Electrolytes, liver function, full blood picture, C-reactive protein, immunoglobulin subclasses and vitamin A levels were within normal range. Commercial western blot revealed anti-retinal antibodies against 36, 44, 60 and 62 kDa retinal proteins. IHC showed only moderate staining of the inner nuclear layer (INL) and ganglion cell layer (GCL) in human retina.

In the absence of evidence of underlying systemic malignancy, the diagnosis of presumed non-paraneoplastic AIR was made. Given the rapid deterioration in vision, the patient agreed to receive 3 doses of pulsed intravenous methylprednisolone followed by slow oral taper over the subsequent 6 months. Rapid improvement in visual symptoms was noted after pulsed steroids, and sustained improvement was confirmed by serial microperimetry over 5 months. However, retinal thinning progressed and paravascular lesions expanded (Online Resource 3).

At 6 months after diagnosis of presumed non-paraneoplastic AIR, the patient presented with urinary obstruction and low back pain. Although serum tumour antigen levels (prostate specific antigen, alpha-fetoprotein, CA19-9 and carcinoembryonic antigen) were within normal limits, C-reactive protein was 256 (normal <10) mg/L and creatinine had risen to 275 (normal: 60–110) µmol/L. CT abdomen and pelvis demonstrated bilateral hydroureter due to obstruction at the level of prostate and bladder, enlarged pelvic lymph nodes and vertebral body fractures at T11 and L2. Nephrostomy was performed, and transurethral biopsy of the prostate showed high-grade small cell neuroendocrine tumour (NET, Online Resource 4). The patient received palliative radiation therapy to spinal metastases and carboplatin/etoposide chemotherapy. He died from metastatic carcinoma in May 2016.

**Fig. 3:**
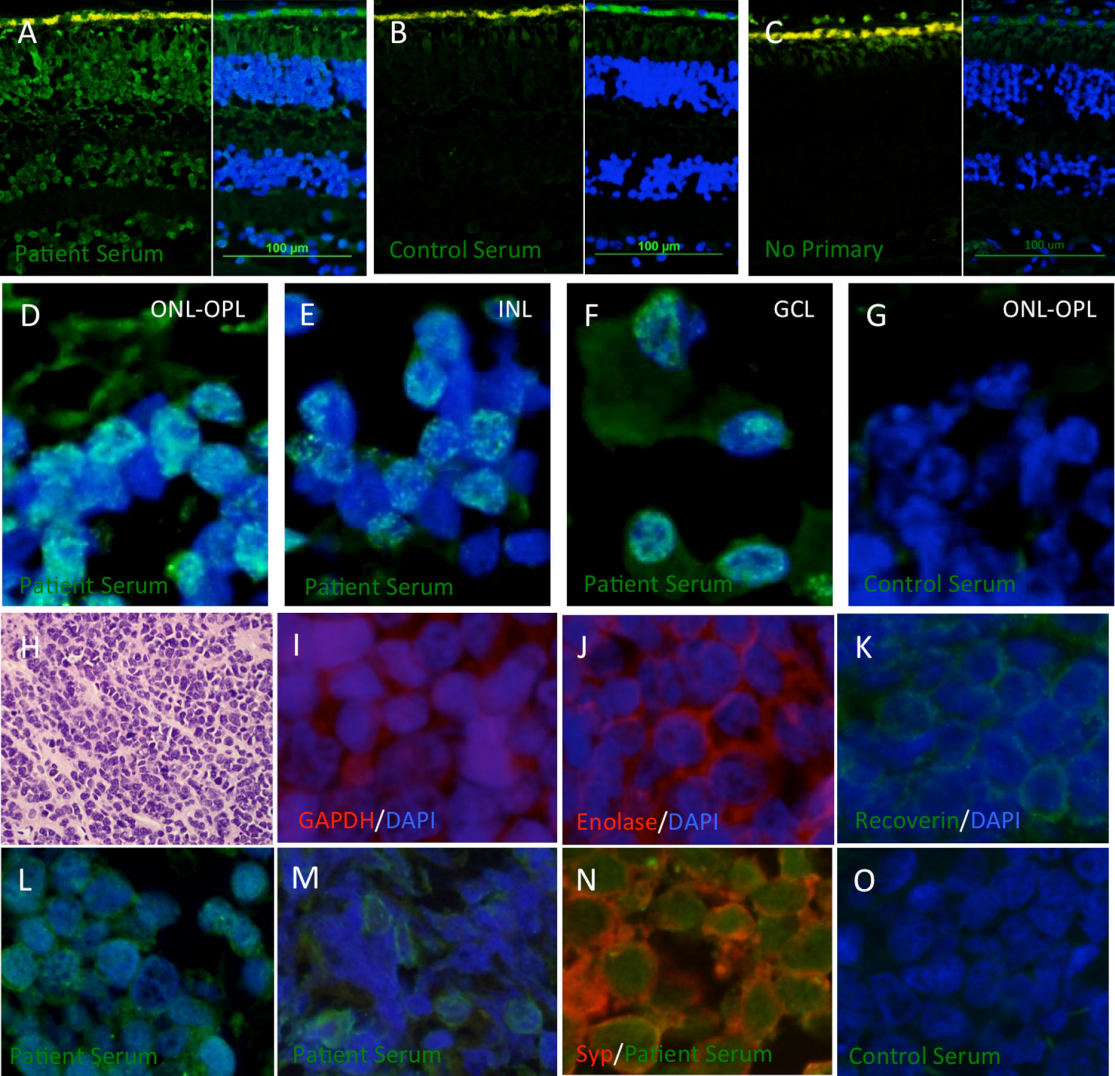
Immunostaining of human retina and prostate tumour sections. **a**–**g** Human retinal IHC was performed using patient serum (**a**, **d**–**f**) or serum from an age- and sex-matched control (**b**, **g**). Negative controls stained with the antihuman IgG-AF488 secondary antibody alone were performed in parallel (**c**). Retinal sections were counterstained for nuclei with DAPI (*blue signal*). Patient IgG antibodies labelled nuclei in the ONL (**d**), INL (**e**) and GCL (**f**, *green signal*). Additional immunolabelling was detected in the OPL (**d**) and in cell bodies of the GCL (**f**). No immunolabelling was detected in retinal sections stained with control serum (**b**, **g**) or in negative controls (**c**) *Scale bars* in **a**–**c** indicate 100 μm. **h**–**o** Patient prostate biopsy sections were examined by H&E staining (**h**) or immunostaining (**i**–**o**). SCC cells displayed immunoreactivity to antibodies specific for GAPDH (**I**), alpha-enolase (**j**), recoverin (**k**) as well as IgG antibodies patient (**l**–**n**) but not control (**o**) serum. Patient serum IgGs labelled synaptophysin positive SCC cells (**n**) and displayed both nuclear (**l**, **n**) and cytoplasmic (**m**) labelling patterns

Our in-house retinal IHC staining showed immunoreactivity of the patient’s serum to nuclear antigens within the ONL, INL and GCL. Weak immunoreactivity was also detected in the outer plexiform layer (OPL) and in the cytoplasm of cells in the GCL (Fig. 3a, d–f). In contrast, IHC using serum from an age- and sex-matched control subject failed to label human retinal sections (Fig. 3b, g).

Immunostaining of prostate tumour sections demonstrated expression of glyceraldehyde-3-phosphate dehydrogenase (GAPDH), recoverin, alpha-enolase and synaptophysin in NET cells (Fig. 3h–k). They also expressed nuclear and perinuclear antigens recognized by immunoglobulin (Ig)-G antibodies present in patient serum, but not in serum from a control subject (Fig. 3l–o).

We further investigated the presence of anti-retinal antibodies in the patient’s serum by western blotting. IgG antibodies present in the patient’s serum labelled multiple bands on western blots containing human retinal protein lysates. In contrast, no significant immunoreactivity was detected in human liver protein lysates (Online Resource 5a). The patient’s serum displayed immunoreactivity to recombinant GAPDH (38 kDa) and alpha-enolase (45 kDa) proteins, but not to paraneoplastic nuclear Ma2 protein (40 kDa) (Online Resource 5b).

## Discussion

We have described the multimodal imaging and immunological features of an acute clinical syndrome characterized by progressive paravascular and placoid neuroretinopathy with negative-type ERG in a patient who subsequently developed NET of the prostate and serum autoantibodies against nuclear and cytoplasmic antigens within the retina.

Previous multimodal imaging studies of established cancer-associated retinopathy (CAR) describe diffuse perimacular outer retinal atrophy on SD-OCT and ring hyper-autofluorescence surrounded by diffuse hypo-autofluorescence closely resembling rod-cone dystrophy [2–7]. In contrast, our case illustrated acute lesions characterized by hyper-reflective infiltrates within the ONL followed rapidly by loss of the EZ and thinning of the ONL. Although these lesions resemble acute macular neuroretinopathy, they were more numerous and extended from the peripapillary region along major vessels. The reduced NIR signal and increased AF within these lesions can be explained by the loss of EZ and photopigment with unmasking of retinal pigment epithelium AF. Although foveal EZ and visual acuity were preserved, mesopic microperimetry demonstrated dense scotoma within the foveal region. The recovery of mesopic sensitivity with systemic steroid suggests that an immune-mediated process against the inner retina may be responsible for visual field loss.

Further support for concurrent inner retinal dysfunction is the presence of negative-type ERG, which is more typically seen in melanoma-associated retinopathy (MAR) although it has also been described in patients with CAR [8–11]. However, these reported CAR cases had no fundus abnormality except for segmental periphlebitis, similar to the typical MAR phenotype. Our case illustrated distinct ERG features that resemble complete congenital stationary night blindness including an essentially flat rod-specific ERG response and square-wave appearance of the single flash photopic ERG due to a delayed and sharply rising b-wave [12]. These features are consistent with depolarizing or ON-bipolar cell dysfunction. The reduced and delayed a-wave suggests additional photoreceptor dysfunction, and therefore, recording of the ON- and OFF-responses to long-flash stimulation is required to conclude that the post-receptoral ON-pathway is more severely affected than the OFF-pathway. Unfortunately, this was not performed on our patient and is a limitation of the ISCEV standard full-field ERG protocol. It is thus a recommendation of this study that future revision of the standard protocol includes long-flash recordings in patients where a negative-type ERG is revealed. Preserved VEP indicated no significant paraneoplastic optic neuropathy. The unusual combination of negative-type ERG and neuroretinopathy on OCT in this case of CAR points to both inner and outer retinopathies. These clinical features are supported by IHC findings, which demonstrated binding of anti-retinal autoantibodies in the patient’s serum to cells in the GCL, INL and ONL of the retina.

The patient’s serum contained IgG antibodies that recognized antigens present in both the prostate NET cells and the human retina (Fig. 3). Western blotting revealed a number of potential antigens present in human retinal protein lysates, two of which were identified as GAPDH and alpha-enolase (Online Resource 5). Both of these antibodies have previously been associated with CAR [13] and were expressed by NET cells in the prostate (Fig. 3i, j). NET cells also expressed the 23-kDa retinal protein recoverin (Fig. 3k). Western blotting of retinal lysates demonstrated patient IgG immunoreactivity to a 23-kDa band. This band was shown to comigrate with recoverin immunoreactivity, suggesting anti-recoverin antibodies may have also been present (Online Resource 5). However, western blotting against a purified recoverin protein is necessary to verify this observation. Together, these results suggest a complex systemic immune response may be recruited against a variety of antigens commonly expressed by retinal and NET cells.

In conclusion, a spectrum of autoreactive antibodies is associated with a unique phenotype of acute progressive paravascular placoid neuroretinopathy resulting in degeneration of photoreceptor cells, suspected dysfunction of depolarizing bipolar cells and classic negative-type ERG in paraneoplastic retinopathy. Detailed clinical, functional and immunological phenotyping of paraneoplastic retinopathy illustrates the complex mechanism of paraneoplastic syndrome.

## Electronic supplementary material

Below is the link to the electronic supplementary material.

Supplementary material 1 (PDF 13493 kb)
