# Supplementary material for: Acute progressive paravascular placoid neuroretinopathy with negative-type electroretinography in paraneoplastic retinopathy
Source: Doc Ophthalmol. 2017 Apr 5;134(3):227–35. doi: 10.1007/s10633-017-9587-9 (PMC5427140; doi:10.1007/s10633-017-9587-9)
Supplement: Supplementary file 1 — Supplementary material 1 (PDF 13493 kb) [file 10633_2017_9587_MOESM1_ESM.pdf]

Acute progressive paravascular placoid neuroretinopathy and retinal depolarizing bipolar cell dysfunction in paraneoplastic retinopathy. Fred K Chen, Avenell L Chew, Dan Zhang, Shang-Chih Chen, Enid Chelva, Erandi Chandrasekera, Eleanor MH Koay, John Forrester, Samuel McLenachan. *Documenta Ophthalmologica*
Email: smclenachan@lei.org.au

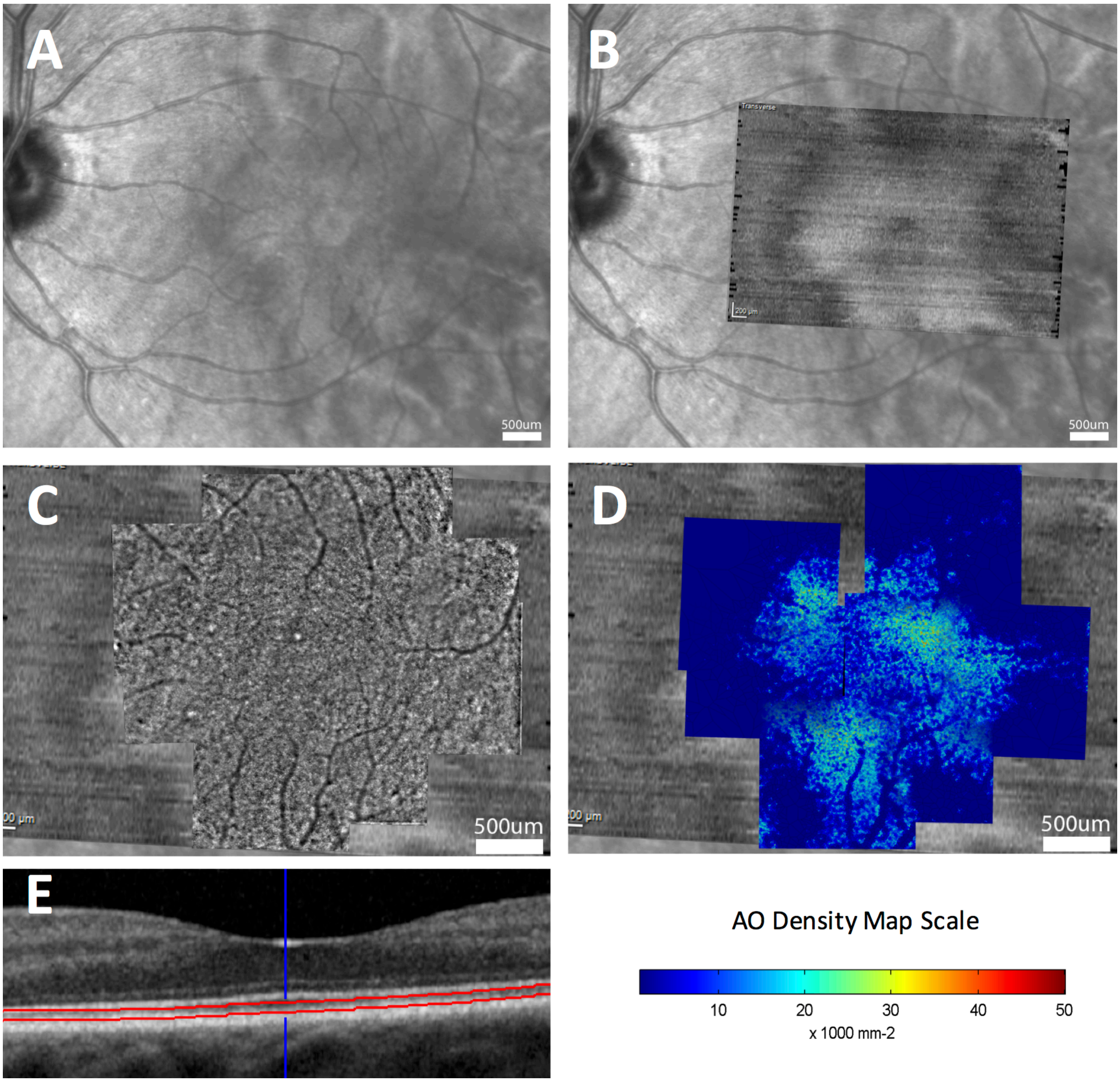

Online Resource 1:

A: Near infrared reflectance (NIR) image of the left eye showed hypo-reflective regions surrounding the central macula. **B:** *En face* reflectivity map of the interdigitation zone overlaid on the NIR image shows preserved signal centrally and loss of signal paracentrally. **C, D:** Adaptive optics cone mosaic and corresponding cone density map show some preservation of cone signal centrally, with marked loss of signal paracentrally in the same pattern that is seen with NIR and interdigitation zone reflectivity maps. **E:** The interdigitaiton zone reflectivity map was generated by manually defining the boundaries of the interdigitation zone on the OCT B-scans using the inbuilt 3D viewing module in the Spectralis software.

Acute progressive paravascular placoid neuroretinopathy and retinal depolarizing bipolar cell dysfunction in paraneoplastic retinopathy. Fred K Chen, Avenell L Chew, Dan Zhang, Shang-Chih Chen, Enid Chelva, Erandi Chandrasekera, Eleanor MH Koay, John Forrester, Samuel McLenachan. *Documenta Ophthalmologica*
Email: smclenachan@lei.org.au

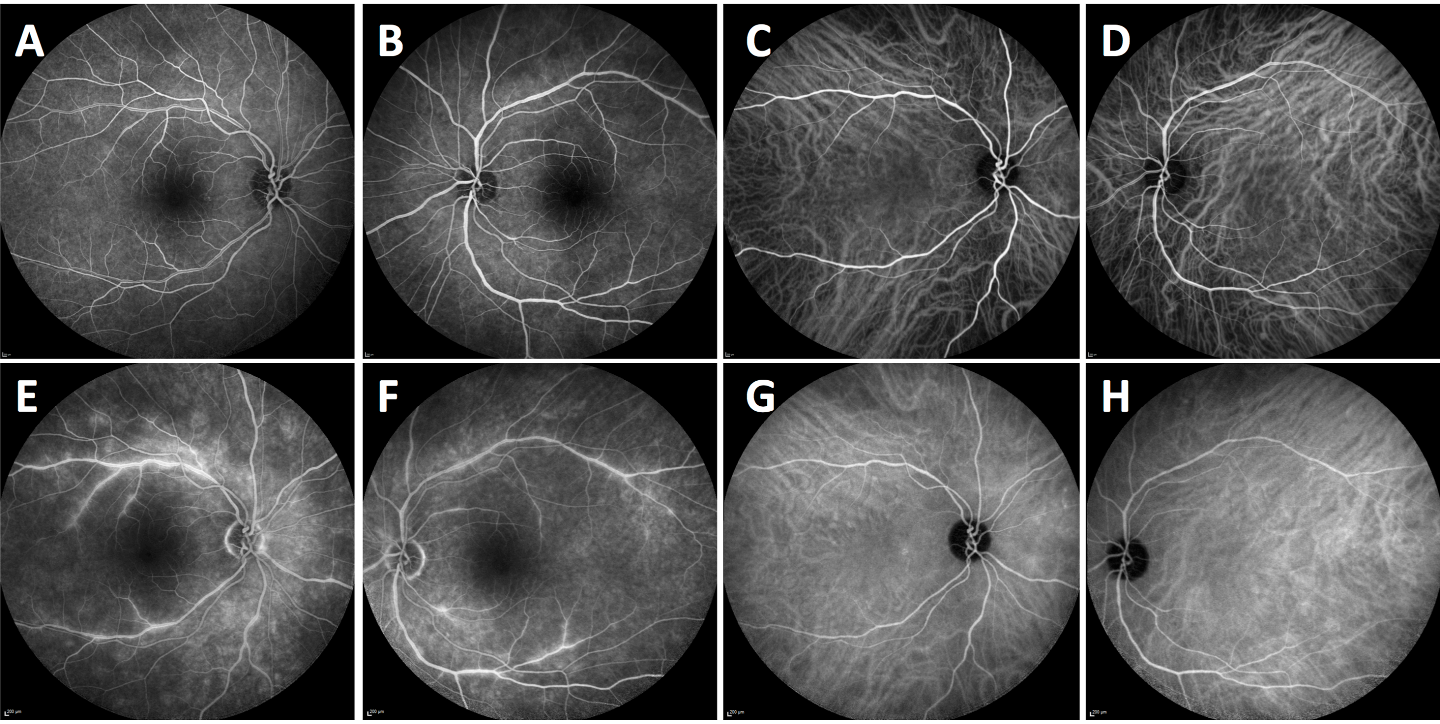

Online Resource 2:

A, B: Early phase fluorescein angiography and **C, D:** indocyanine angiography were normal in the early phase. **E, F:** Late phase fluorescein angiography showed staining in the paravascular regions and segmental leakage along the retinal veins. **G, H:** Late phase indocyanine angiography remained normal.

Acute progressive paravascular placoid neuroretinopathy and retinal depolarizing bipolar cell dysfunction in paraneoplastic retinopathy. Fred K Chen, Avenell L Chew, Dan Zhang, Shang-Chih Chen, Enid Chelva, Erandi Chandrasekera, Eleanor MH Koay, John Forrester, Samuel McLenachan. *Documenta Ophthalmologica*

Email: smclenachan@lei.org.au

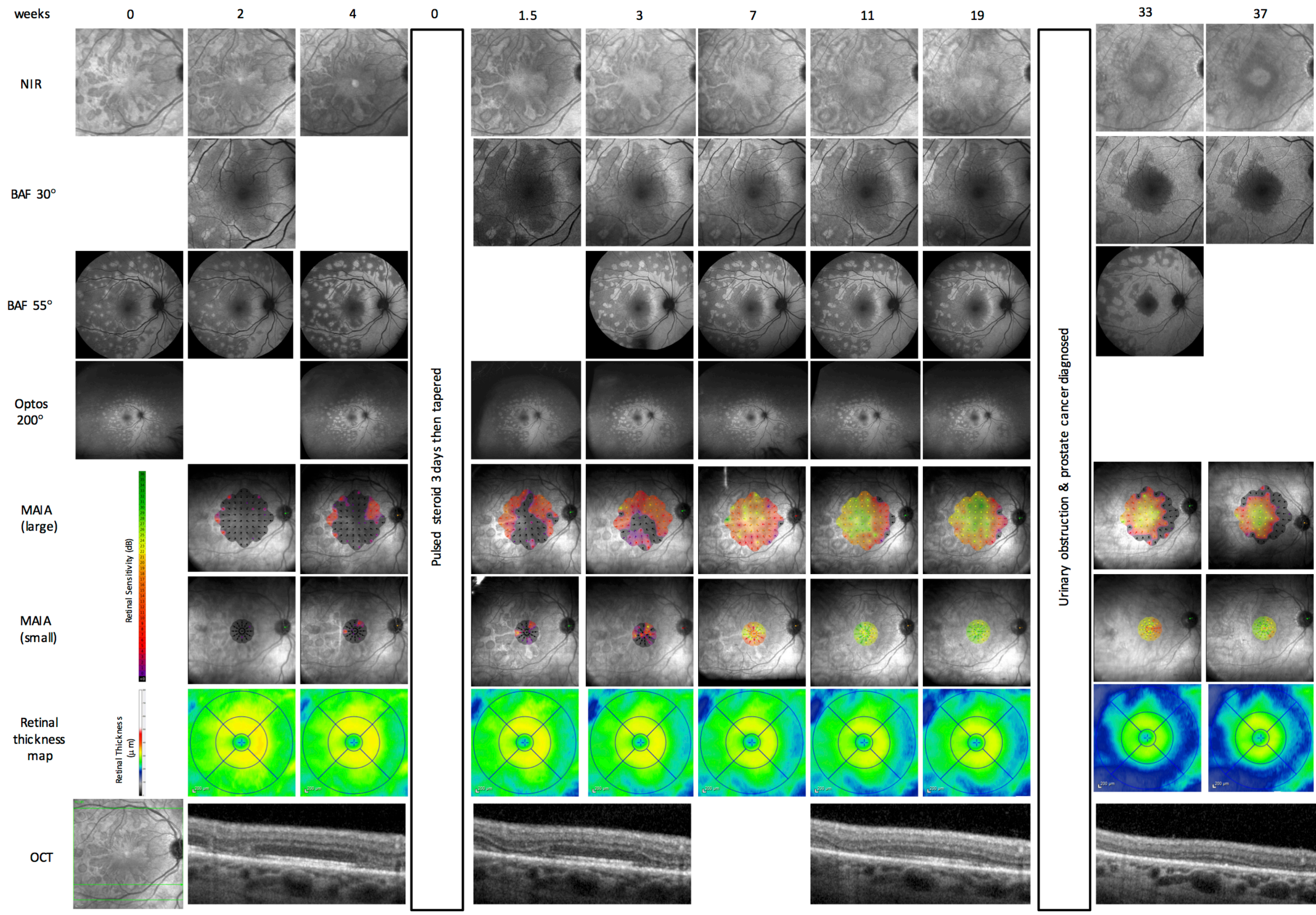

Online Resource 3:

Near infrared reflectance shows discrete hyporeflective lesions that increase in size and coalesce over the follow up period, with continuing expansion of the lesions despite treatment with steroids. The same pattern of progression is observed in the blue autofluorescence and green autofluorescence (BAF, GAF) (optos 200°) images, with progressive expansion of areas of unmasked autofluorescence over time. Macular sensitivities on microperimetry (MAIA) improved dramatically after treatment with pulsed steroids, before declining towards the end of follow up. There was progressive thinning of the retina on OCT despite steroids.

Acute progressive paravascular placoid neuroretinopathy and retinal depolarizing bipolar cell dysfunction in paraneoplastic retinopathy. Fred K Chen, Avenell L Chew, Dan Zhang, Shang-Chih Chen, Enid Chelva, Erandi Chandrasekera, Eleanor MH Koay, John Forrester, Samuel McLenachan. *Documenta Ophthalmologica*
Email: smclenachan@lei.org.au

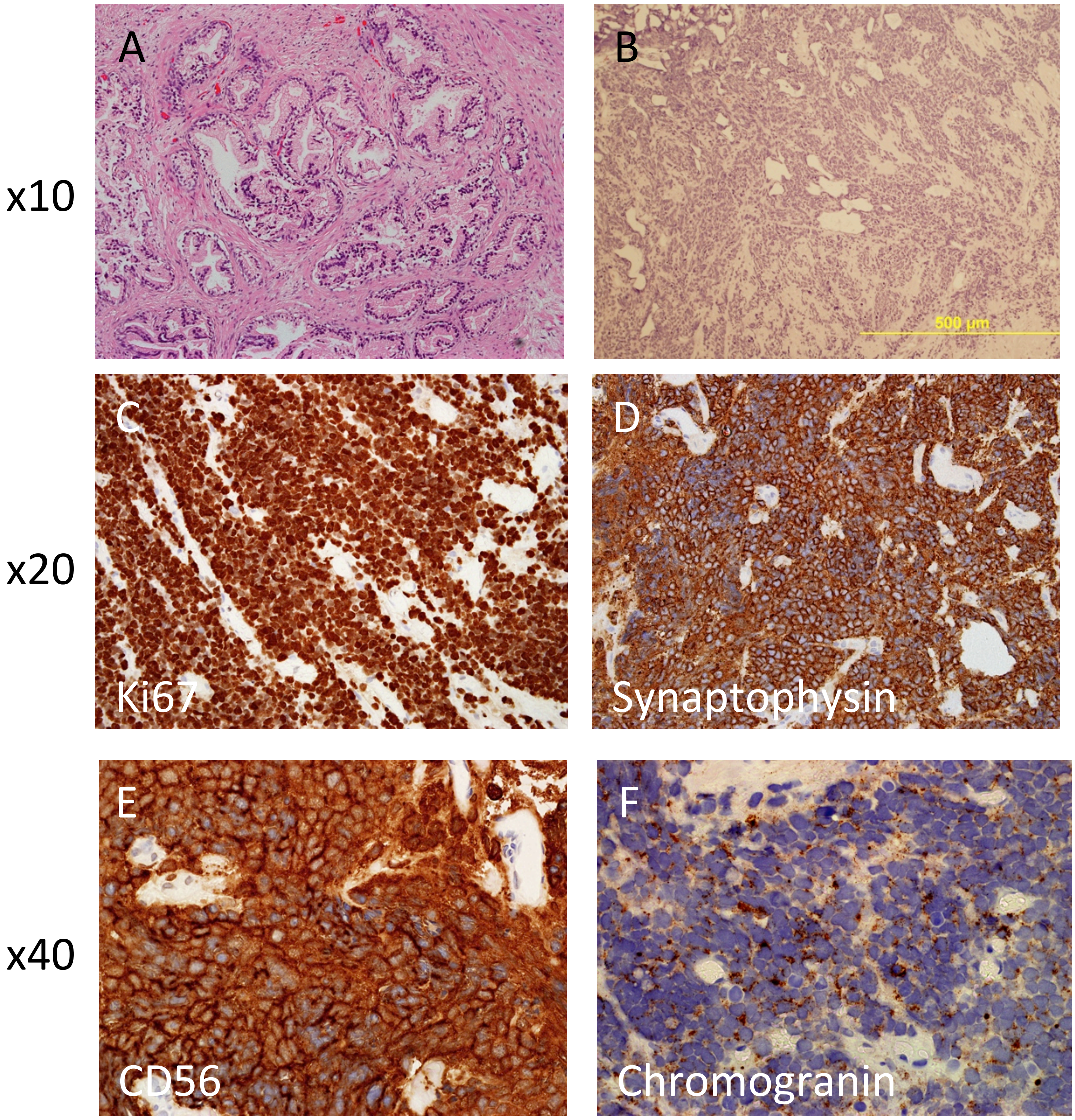

Online Resource 4:
A: An H&E stained prostate biopsy section from a control subject demonstrating normal prostate histology. **B-F:** Sections from the patient's prostate biopsy revealed widespread small cell carcinoma (SCC) (**B**). SCC cells were immunopositive for the proliferation marker Ki67 (**C**), and the neuroendocrine markers synaptophysin, CD56 (**D**) and chromogranin (**F**), consistent with neuroendocrine malignancy.

Acute progressive paravascular placoid neuroretinopathy and retinal depolarizing bipolar cell dysfunction in paraneoplastic retinopathy. Fred K Chen, Avenell L Chew, Dan Zhang, Shang-Chih Chen, Enid Chelva, Erandi Chandrasekera, Eleanor MH Koay, John Forrester, Samuel McLenachan. *Documenta Ophthalmologica*
Email: smclenachan@lei.org.au

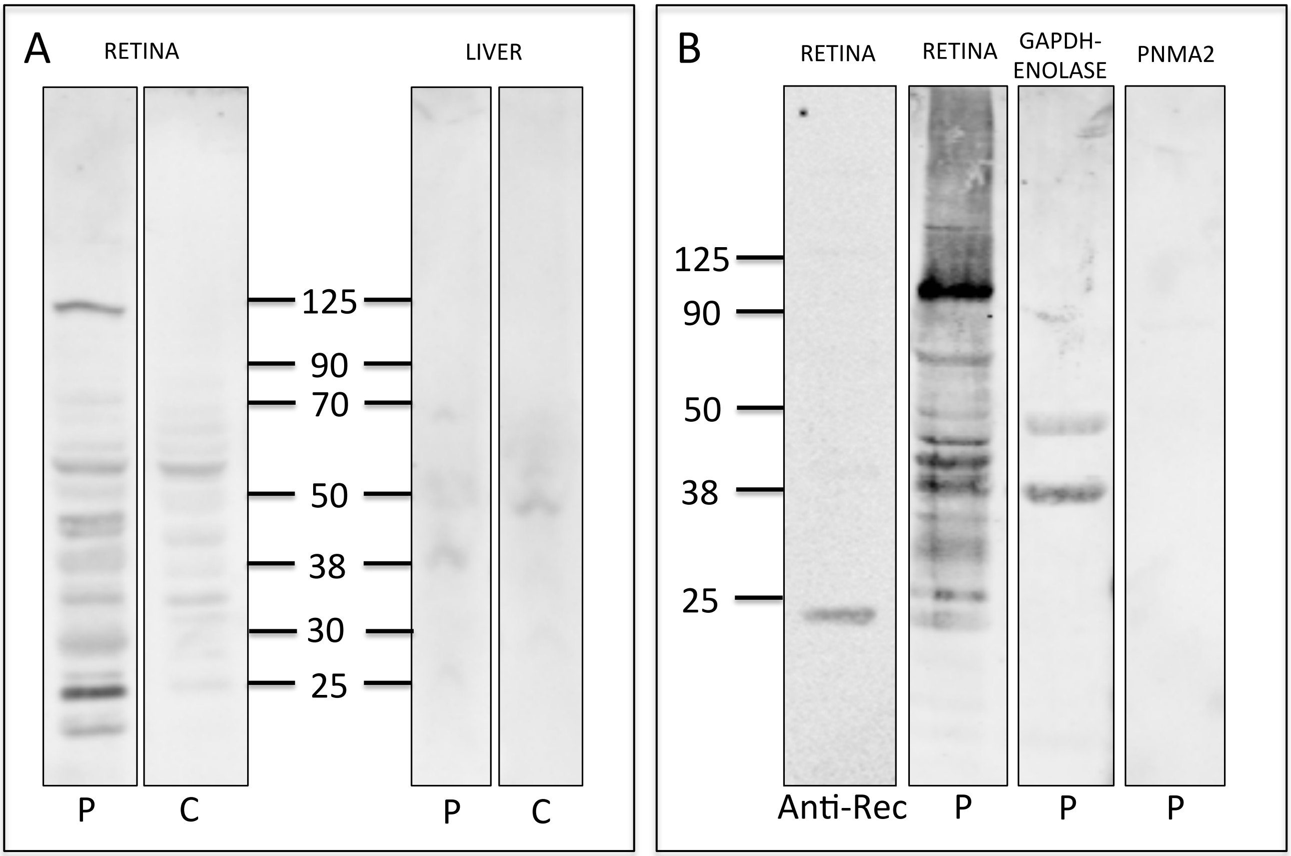

Online Resource 5:

A: Western blot showing patient (P) and control (C) serum IgG labelled retinal (left lanes) and liver (right lanes) protein lysates. Band sizes are indicated in kDa. **B:** Western blots performed using retinal lysates (1st and 2nd lanes) and recombinant proteins (3rd and 4th lanes). Anti-recoverin antibody (Anti-Rec) labelled a single 23 kDa band in retinal protein lysates, corresponding to a 23 kDa band labelled by patient serum IgG antibodies (P). Patient serum IgG antibodies labelled both GAPDH (38 kDa) and alpha enolase (45 kDa) in a pooled sample of both recombinant proteins (3rd lane), but not recombinant PNMA2 protein (4th lane). Band sizes are indicated in kDa.
